# The complete chloroplast genome sequence of *Spiraea blumei* G. Don (Rosaceae)

**DOI:** 10.1080/23802359.2019.1678434

**Published:** 2019-10-21

**Authors:** Yan Huo, Ming Yan, Xueqing Zhao, Zunling Zhu, Zhaohe Yuan

**Affiliations:** a College of Landscape Architecture, Co-Innovation Center for Sustainable Forestry in Southern China, Nanjing Forestry University, Nanjing, China;; b College of Forestry, Nanjing Forestry University, Nanjing, PR China;; c College of Arts and Design, Nanjing Forestry University, Nanjing, PR China

**Keywords:** *Spiraea blumei*, complete chloroplast genome, phylogenetic analysis

## Abstract

*Spiraea blumei* G. Don is an ornamental shrub widely distributed in Eastern Asia. Here, we reported and characterized the complete chloroplast (cp) genome sequence of *S. blumei* (GenBank accession number: MN418904) to provide genomic resources for promoting its conservation. The total chloroplast genome is 155,957 bp in length and contains the typical chloroplast structure, including two inverted repeat (IR) regions of 26,343 bp, a large single-copy (LSC) region of 84,384 bp, and a small single-copy (SSC) region of 18,887 bp. The overall guanine-cytosine (GC) content of the *S. blumei* chloroplast genome is 36.8%. The cp genome encodes 133 unique genes, including 85 protein-coding genes (PCGs), 40 tRNA genes, and 8 rRNA genes. We used the cp genome of *S. blumei* and 26 other cp genomes to perform a phylogenetic analysis, which indicated that *S. blumei* was closely related to *S. martini* in Rosaceae.

*Spiraea blumei* G. Don is a plant of the family Rosaceae and widely distributed in China, Japan, and Korea. It has high ornamental value for its white early summer flowers and elegant leaves (Zhang 2010). Besides, its leaves are a tea substitute (Kunkel 1984). So far, there have been no studies on the genome of *S. blumei*. Therefore, the genetic and genomic information is urgently needed to promote its conservation and systematics research. In this study, we sequenced the complete chloroplast (cp) genome of *S. blumei* based on an Illumina platform and analysed genetic relationships with the closely related species in Rosaceae.

Experimental samples were collected from Nanjing, China (latitude: 32°07′86.1ʺN, longitude: 118°81′69.4ʺ E) and deposited at Nanjing Forestry University (accession number NFU19051103). Total DNA was extracted from fresh leaves of *S. blumei* individual by the modified method CTAB (Doyle 1987). Paired-end libraries were constructed and sequenced with an Illumina Hiseq 2500 platform (Nanjing, China) for paired-end 150 bp reads. In total, ca. 83.1 million high-quality clean reads were generated with adaptors trimmed. Then, NOVOPlasty3.1 (Dierckxsens et al. 2017) was used for *de novo* assembly with *Prunus yedoensis* (GenBank accession KU985054) as a reference. GeSeq (Tillich et al. 2017) was used for annotation and Geneious version 8.0.4 (Kearse et al. 2012) was used for inspecting and characterizing the chloroplast genome.

The complete cp genome size of *S. blumei* is 155,957 bp in length, containing the large single copy (LSC, 84,384 bp), a small single copy (SSC, 18,887 bp), and two inverted repeats (IR, 26,343 pb) regions. The overall GC content of *S. blumei* cp genome is 36.8% and those in the LSC, SSC, and IR regions are 34.6%, 30.4%, and 42.5%, respectively. The cp genome encodes 133 unique genes, including 85 protein-coding genes (PCGs), 40 tRNA genes, and 8 rRNA genes. The tRNA genes are distributed throughout the whole genome with 25 in the LSC, 1 in the SSC, 14 in the IR regions, while rRNAs only situate in IR regions. Twenty-one genes have two copies, which include eight PCGs (*ndhB*, *rpl2*, *rpl23*, *rps12*, *rps19*, *rps7*, *ycf1*, *ycf2*), nine tRNA genes (*trnA-UGC*, *trnI-CAU*, *trnI-GAU*, *trnL-CAA*, *trnM-CAU*, *trnN-GUU*, *trnR-AC*G, trnT-GGU, trnV-GAC), and four all rRNA genes *(rrn16*, *rrn23*, *rrn4.5*, and *rrn5*). Among the PCGs, two genes (*ycf3* and *rps12*) contain two introns, and 13 different genes (*atpF*, *clpP*, *ndhA*, *ndhB*, *rpl2*, *rpoC1*, *rps16*, *trnA-UGC*, *trnG-UCC*, *trnI-GAU*, *trnK-UUU*, *trnL-UAA*, *trnV-UAC*) have one intron each. The *rps12* is a trans-spliced gene with 5′ end located in the LSC region and the duplicated 3′ end in the IR regions.

Twenty-seven chloroplast genomes sequences were aligned with MAFFT version 7.3 (Katoh and Standley 2013), and the maximum likelihood (ML) inference was performed using TVM + F+R3 model with 1000 bootstrap replicates *via* IQ-TREE version.1.6.8 (Nguyen et al. 2015). The result revealed that *S. blumei* is closer to *S. martini* in Rosaceae (Figure 1). The complete chloroplast sequence of *S. blumei* will provide useful information for future studies to solve the problems related to the phylogeny of genus *Spiraea* and family Rosaceae.

**Figure 1.**
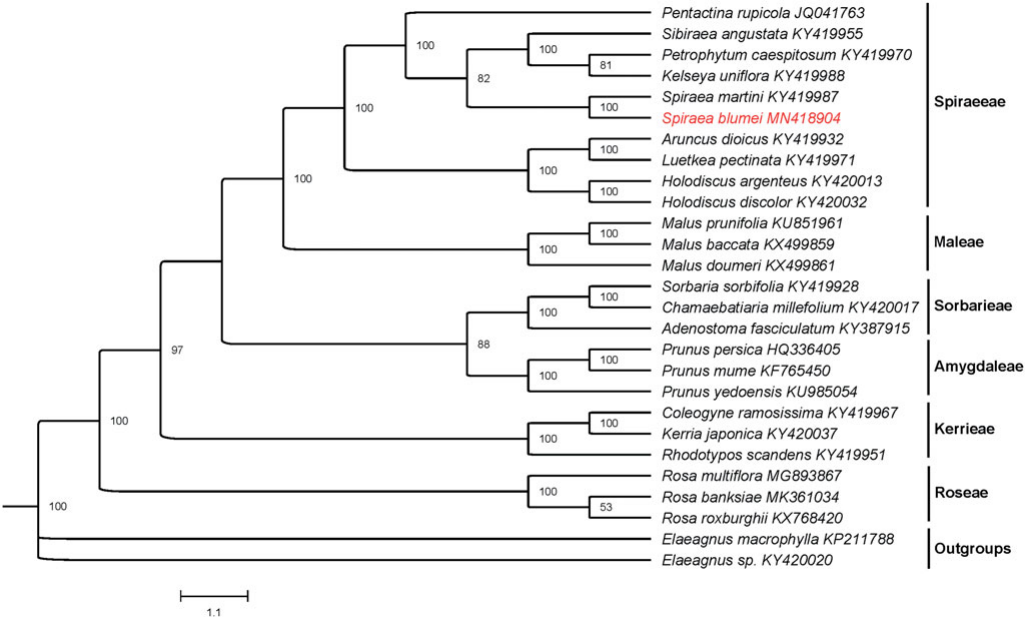
Maximum likelihood (ML) tree based on the chloroplast genome sequences of 27 species. Numbers on the nodes are bootstrap values from 1000 replicates. *Elaeagnus macrophylla* and *Elaeagnus* sp. were selected as outgroups.
